# Prevalence and genetic diversity of rotavirus in Bangladesh during pre-vaccination period, 1973-2023: a meta-analysis

**DOI:** 10.3389/fimmu.2023.1289032

**Published:** 2023-11-22

**Authors:** Nadim Sharif, Nazmul Sharif, Afsana Khan, Irma Domínguez Azpíroz, Raquel Martínez Diaz, Isabel De la Torre Díez, Anowar Khasru Parvez, Shuvra Kanti Dey

**Affiliations:** ^1^ Department of Microbiology, Jahangirnagar University, Dhaka, Bangladesh; ^2^ Department of Mathematics, Rajshahi University of Engineering & Technology, Rajshahi, Bangladesh; ^3^ Department of Statistics, Jahangirnagar University, Dhaka, Bangladesh; ^4^ Universidad Europea del Atlántico, Santander, Spain; ^5^ Universidad Internacional Iberoamericana, Arecibo, PR, United States; ^6^ Universidad de La Romana, La Romana, Dominican Republic; ^7^ Universidade Internacional do Cuanza, Cuito, Bié, Angola; ^8^ Universidad Internacional Iberoamericana, Campeche, Mexico; ^9^ University of Valladolid, Valladolid, Spain

**Keywords:** rotavirus, genotype, epidemiology, diversity, Bangladesh, pre-vaccination

## Abstract

**Introduction:**

Rotavirus infection is a major cause of mortality among children under 5 years in Bangladesh. There is lack of integrated studies on rotavirus prevalence and genetic diversity during 1973 to 2023 in Bangladesh.

**Methods:**

This meta-analysis was conducted to determine the prevalence, genotypic diversity and seasonal distribution of rotavirus during pre-vaccination period in Bangladesh. This study included published articles on rotavirus A, rotavirus B and rotavirus C. We used Medline, Scopus and Google Scholar for published articles. Selected literatures were published between 1973 to 2023.

**Results:**

This study detected 12431 research articles published on rotavirus. Based on the inclusion criteria, 29 of 75 (30.2%) studies were selected. Molecular epidemiological data was taken from 29 articles, prevalence data from 29 articles, and clinical symptoms from 19 articles. The pooled prevalence of rotavirus was 30.1% (95% CI: 22%-45%, *p* = 0.005). Rotavirus G1 (27.1%, 2228 of 8219) was the most prevalent followed by G2 (21.09%, 1733 of 8219), G4 (11.58%, 952 of 8219), G9 (9.37%, 770 of 8219), G12 (8.48%, 697 of 8219), and G3 (2.79%, 229 of 8219), respectively. Genotype P[8] (40.6%, 2548 of 6274) was the most prevalent followed by P[4] (12.4%, 777 of 6274) and P[6] (6.4%, 400 of 6274), respectively. Rotavirus G1P[8] (19%) was the most frequent followed by G2P [4] (9.4%), G12P[8] (7.2%), and G9P[8], respectively. Rotavirus infection had higher odds of occurrence during December and February (aOR: 2.86, 95% CI: 2.43-3.6, *p* = 0.001).

**Discussion:**

This is the first meta-analysis including all the studies on prevalence, molecular epidemiology, and genetic diversity of rotavirus from 1973 to 2023, pre-vaccination period in Bangladesh. This study will provide overall scenario of rotavirus genetic diversity and seasonality during pre-vaccination period and aids in policy making for rotavirus vaccination program in Bangladesh. This work will add valuable knowledge for vaccination against rotavirus and compare the data after starting vaccination in Bangladesh.

## Introduction

Group A rotavirus is the main (family *Reoviridae*) pathogen of gastroenteritis among children under five years in Bangladesh (1–3). About 300 000 deaths related to rotavirus infection are reported worldwide every year (1). Rotavirus associated death has been reduced in the developed countries due to the implementation of rotavirus vaccination program (4–6). However, the health burden of rotavirus associated morbidity and mortality are still high in developing countries like Bangladesh without vaccination against rotavirus (7–9). The estimated incidence of rotavirus is 10,000 cases per 100,000 children under five years in Bangladesh. According to WHO, severe health conditions are reported from 25-30% of rotavirus infected children (8, 9). Every year about 2500-3000 children under five die of rotavirus infection in Bangladesh. The TRIVAC model by Pan American Health Organization indicates that vaccination against rotavirus can prevent about 3 million cases and 4000 fatalities among children under five during next 10 years in Bangladesh (7–10).

Rotavirus is a nonenveloped, double stranded RNA (dsRNA, segmented) virus with a genome of about 18.55 kilo base pairs (11). Rotavirus encodes for six structural proteins namely, VP1 to VP6 and six nonstructural proteins namely, NSP1 to NSP6 (10, 11). Among ten species of rotavirus (referred to as A, B, C, D, E, F, G, H, I and J), rotavirus A is mainly involved with majority of human cases (90%). Two genotypes of rotavirus are named as G genotype from glycoprotein (VP7) and P genotype from protease (VP4) (11, 12). Nearly, 36-types and 51 P-types have been reported worldwide since 1973 (10). Until 2000s, the most commonly reported G-types were G1‐G4 (11–17). After 2000s, genotype G9 transmitted rapidly became one of important genotypes worldwide, whereas genotype G12 became prevalent in the Americas and developing countries in Asia. Among the P-type, P[4], P[6] and P[8] remain the most common worldwide (12–17). Globally rotavirus genotypes G1P[8], G2P[4], G3P[8], G4P[8] and G9P[8] have contributed to the majority of the reported cases.^(12-17)^ The genotypic diversity of rotavirus has changed in Bangladesh over the years (16, 17).

Four rotavirus A vaccines are prequalified by WHO and available internationally namely, RotaTeq (bovine-human reassorted vaccine), Rotarix (human rotavirus), Rotavac (bovine-human reassorted vaccine of G9P11), and RotaSiil (bovine-human reassorted vaccine) Besides, several other vaccines are available and licensed for use in India (Rotasiil, pentavalent human bovine reassortant vaccine), Vietnam (Rotavin-M1, human genotyp G1P[8]) and China (Lanzhou lamb, genotype G10P[12]of lamb rotavirus) (10). Though rotavirus vaccination is not included in the national immunization program in Bangladesh, international rotavirus vaccines are available in Bangladesh (9, 10, 13). The efficacy of rotavirus vaccines namely, Rotarix and RotaTeq is high in developed countries (13). After the introduction of rotavirus vaccination, the cases and fatalities among children under five reduced significantly in the developed countries. However, in developing and under-developed countries the vaccines have lower efficacy. A recent Rotarix trial in Bangladesh has revealed that vaccine efficacy is decreasing and it is below 50%. This lower efficacy may be attributed to the circulation of heterotypic strains and high number of mutational and reassortment events of homotypic rotaviruses (9, 10). However, integrated studies on the prevalence and epidemiology of rotavirus are lacking in Bangladesh. Knowing the epidemiology and genetic diversity of rotavirus can contribute in evaluation of effects of rotavirus vaccines in Bangladesh. The main aim of this study is to determine the prevalence and molecular epidemiology of rotavirus in Bangladesh during pre-vaccination period, from 1973 to 2023.

## Methods

### Definitions

Epidemiology of rotavirus is defined as the distribution and determinants of the outbreaks in different population and strategies taken to minimize the health effect among them. The clinical feature was defined including both the signs and symptoms during and after the rotavirus infection. Transmission of rotavirus is defined as the transfer of the virus from source to human and carrier to human. This study included epidemiological, clinical and virological research published in Bangladesh on rotavirus. The rotavirus positive case was determined by ELISA-based method before the invention of RT-PCR laboratory-confirmed RT-PCR test. The standards of the Preferred Reporting Items for Systematic Review and Meta-Analysis (PRISMA) Statement and Cochrane Collaboration were followed to conduct the study (18).

### Study design

The study was performed by following different steps including, identification of precise objectives and search strategies, appropriate research articles, inclusion of manuscripts, collection of data, analysis, and summarization and inclusion of the findings, exclusion of irrelevant studies. We included previous original research articles focusing epidemiological studies, case studies, surveillance work, outbreak investigation, hospital-based surveillance and online databases. No previous study on the topic with strict parameters for the quality assessment is available. As a result, we relied on the quality reports of the selected articles by the authors.

### Search strategy and selection criteria

We performed searches for collecting relevant articles in MEDLINE (through PubMed), Web of Science, EMBASE, Scopus, The New England Journal of Medicine (NEJM) and The Lancet with no restriction on language. The place of the search strategy was fixed for Bangladesh. All published manuscripts and scientific writings till December 31, 2022 were included in this study. The notable search term included Rotavirus, RVA, RVB, RVC, Epidemiology of rotavirus, Epidemiology of rotavirus in Bangladesh, Clinical features of rotavirus, Sign and Symptoms of rotavirus, Clinical characteristics of rotavirus, Cases of rotavirus, Transmission of rotavirus, Molecular epidemiology of rotavirus, Genetic diversity, Bangladesh, Children under five, and combination of these search terms. Distinguish searches were made for each term in every website and database.

Further, we performed search on the grey literature and Google Scholar database. These sources included data and articles from CDC (Centers for Disease Control and Prevention, USA), ECDC (European Centers for Disease Control and Prevention) WHO (the World Health Organization), Epicentre, ProMed, IEDCR of Bangladesh, ICDDR, B in Bangladesh. We searched for the yearly update of the data in these websites and databases. Further, articles and data were searched and included from different preprint databases including SSRN, bioRxiv, medRxiv, and AAS Open Research. Additionally, we manually analyzed the first twenty pages on the Google Scholar search system for each search term. We tried to determine the epidemiology of rotavirus by including prevalence over the years, genetic diversity, incidence, case reports, clinical history, case fatality rate, seasonality and vaccination.

Two authors, NS and NS conducted the evaluation of eligible scientific studies. After completion of searching of all the databases and websites, the potential articles were selected by removing the duplicates and screening out the eligible ones by NS and AKP. Articles with specific and relevant topics were selected for all cities and location in Bangladesh, all ethnicity, age group, sex and clinical features for full-text analyzing. We also included the articles focusing on the molecular epidemiology of rotavirus involving animals and human or only animal or only human published during 1973 and 2022. We excluded modelling and prediction studies, correlation of environmental factors studies, review articles and non-relevant studies to our objectives. Further, critical evaluation on the quality of the selected articles, screening out duplicated articles, and removing correspondence or comment of duplicated data were performed by NS, SKD and AK, separately. The seasonal exclusion criteria were implemented only on the studies on seasonality of rotavirus not on environmental impact.

The risk of bias was measured using The Systematic Review Centre for Laboratory Animal Experimentation (SYRCLE) assessment tool (19). The SYRCLE is consisted of 10 parameters to assess different biases in this study. The parameter included attrition bias, detection bias, reporting bias, performance bias, selection bias and other biases. The bias for each parameter was measured by using the possible outcomes as yes, no, and unclear, representing low, high, and unclear bias, respectively (19). Data on environmental factors were collected from different websites including, the official website of Bangladesh Meteorological Department (http://live4.bmd.gov.bd/satelite/v/sat_infrared/), meteoblue (www.meteoblue.com), AccuWeather (www.accuweather.com) and WeatherOnline (www.weatheronline.co.uk). Data on sociodemographic factors were included from the analyzed studies and database of national surveillance system on rotavirus in Bangladesh.

### Case definition

A confirmed case of rotavirus was defined by following the WHO case definition and final classification guideline, the presence of rotavirus is confirmed by an enzyme immunoassay (EIA) or reverse transcriptase polymerase chain reaction (PCR)-based methods. The type of the used PCR assay will be determined on the basis of local guidelines and availability of the test method.

### Statistical analysis

Total number of cases, circulating genotypes and seasonality were determined for each decade by summation of the reported cases per genotypes. Pooled statistical analyses were conducted for determining the overall prevalence of rotavirus and genotypic distribution by using SAS version 9.4. Logistic regression analysis was conducted to determine the impact of sociodemographic factors and environmental factors on the odds of rotavirus infection.

## Results

### Studies analyzed and included

**Figure 1:**
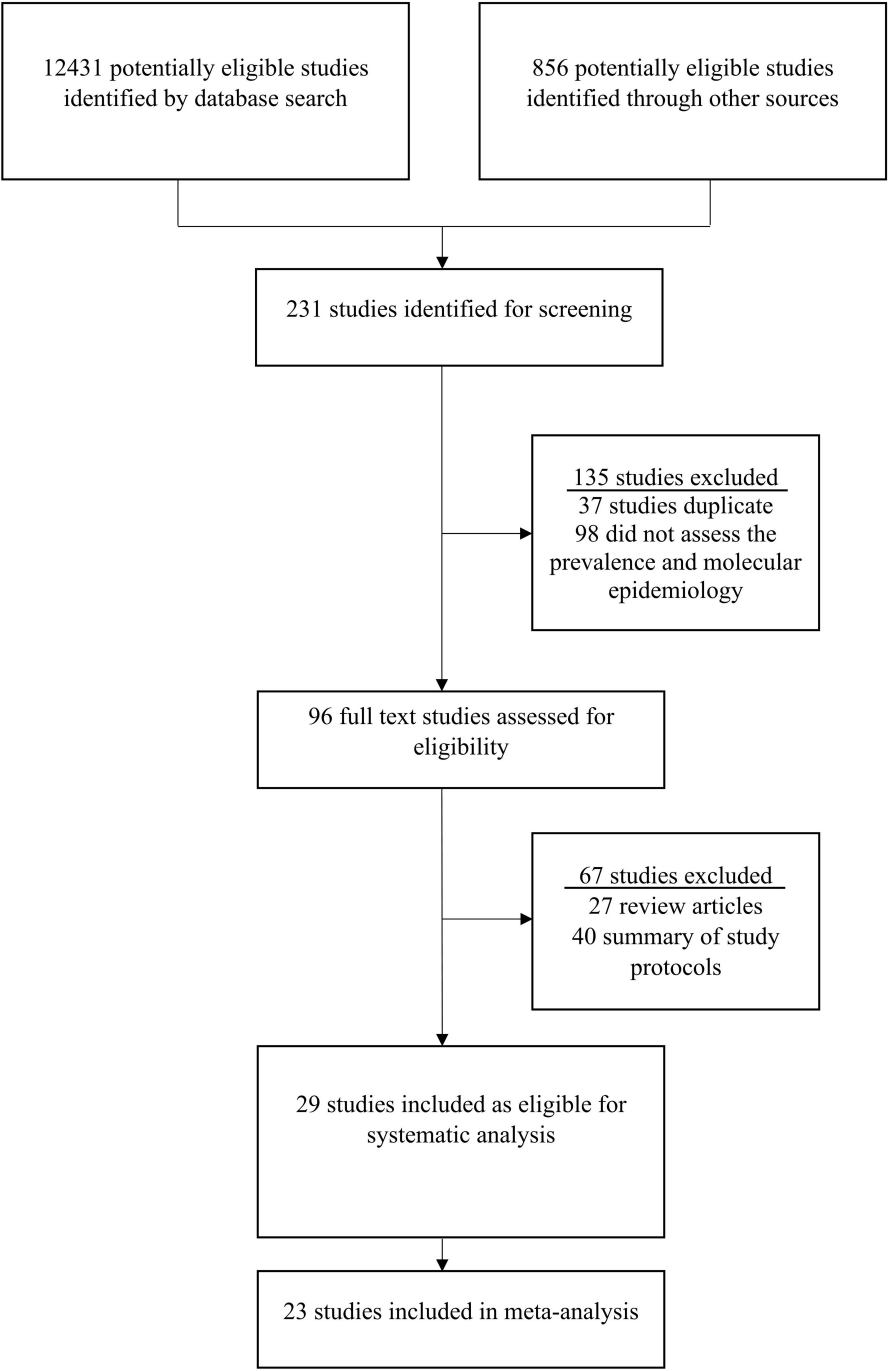
Procedure of study selection on rotavirus in Bangladesh. The standards of the Preferred Reporting Items for Systematic Review and Meta-Analysis (PRISMA) Statement was followed to select the studies.

This study detected 12431 research articles on the prevalence and molecular epidemiology of rotavirus in Bangladesh (**Figure 1**). Firstly, among 12431 articles, 231 were screened and initially considered for analyzing abstract. Secondly, from 231 articles we selected 96 articles for full text investigation and excluded 135 articles. Thirdly, after analyzing the full texts, we excluded 67 studies and included 29 (30.2%) studies for further analysis. Among the selected manuscripts, we extracted molecular epidemiological data from 29 articles, prevalence data from 29 articles, clinical symptoms from 19 articles (**Figure 1**). Finally, 23 studies were included in the meta-analysis.

### Pooled prevalence of rotavirus infection

Prevalence of rotavirus infection varied from the first report to the last report of rotavirus during the last 50 years in Bangladesh. The pooled prevalence of rotavirus infection was 30.1% (95% CI: 22%-45%, *p* = 0.005) in Bangladesh before the introduction of mass vaccination. Among these 29 studies during 1973-2023, rotavirus infection was reported from 25 (86.2%) studies (20–50). Rotavirus A (23 of 25, 92% studies) was the most predominant followed by rotavirus B (1 of 25, 4% study) and C (1 of 25, 4% study). Rotavirus A was reported from about 99.93% (45671 of 45679) of the cases (**Table 1**). Most of the cases (80%) were reported among children. Co-prevalence of pathogenic bacteria, virus and parasites were reported in 7 studies. Male was the most commonly affected group. In majority of the studies, ELISA and reverse-transcriptase polymerase chain reaction was used to detect the presence of rotavirus (**Table 1**). Before 2010, studies on rotavirus were conducted in only three cities including Dhaka, Mymensingh and Matlab.

**Table 1:** Prevalence and epidemiology of rotavirus infection in Bangladesh, 1973-2023.

Study	Year/Place	No.	Age	Sex ratio Male: Female	Rota positive number (%)	Diagnosis method	Species	Other pathogens (%)
Black et al. (1981) (20)	1977-79, Matlab	877	<2 years	N/A	43 (5)	ELISA	RVA	*E. coli* (23) *Shigella* spp (11)
Black et al. (1980) (21)	1978-79, Matlab	6352	All	N/A	1525 (24)	ELISA	RVA	*E. coli* (32), *Salmonella* (<1), *Shigella* (6), V*. cholerae* (14), *V. parahaemolyticus* (<1)
Ahmed et al. (1989) (22)	1988, Dhaka, Mymensingh	414	All	N/A	124 (30)	ELISA PAGE	RVA	N/A
Tabassum et al. (1989) (23)	Dhaka	111	<5 years	N/A	35 (32)	ELISA PAGE RT-PCR	RVA	N/A
Bingnan et al. (1991) (24)	1987-89, Matlab	5811	All	N/A	898 (15.5)	ELISA PAGE	RVA	*Shigella* spp
Berne et al. (1992) (25)	1987-89, Matlab	2441	<2 years	N/A	764 (31)	ELISA	RVA	N/A
Sanekata et al. (2003) (26)	2000-01, Mymensingh	287	All	N/A	14 (4.9)	RT-PCR	RVB	N/A
Rahman et al. (2005) (27)	2003, Dhaka	611	All	1.3:1	14 (2.3)	RT-PCR	RVC	*Vibrio cholerae* O1 (8) *S. flexneri* (2)
Rahman et al. (2007) (28)	2001-2006, Total	19,039	All	N/A	4,712 (24)	Dakopatts kit RT-PCR	RVA	N/A
	Dhaka	10739			2706 (25.2)			
	Matlab	8300			1938 (23.3)			
Tanaka et al. (2007) (29)	1993-2004, Matlab	4596	All	N/A	1617 (35)	ELISA	RVA	*V. cholerae* (6) *Shigella* (11) *Salmonella* (1) Parasites (2)
	2000-2004, Dhaka	18,300			6056 (33)			*V. cholerae* (12) *Shigella* (7) *Salmonella* (2) Parasites (2)
Paul et al. (2008) (30)	2004-2006, Mymensingh	1627	Children	N/A	430 (26.4)	RT-PCR	RVA	N/A
		913	Adult		92 (10.1)			
Paul et al. (2008) (31)	2004-2006, Mymensingh	1438	All	N/A	171 (11.9)	ELISA	RVA	N/A
Zaman et al. (2009) (32)	2000-2006, Matlab	4519	<5 years	N/A	1479 (33)	ELISA RT-PCR	RVA	N/A
Dey et al. (2009) (33)	2004-2005, Dhaka	917	<5 years	1.3:1	307 (33.5)	RT-PCR	RVA	N/A
Islam et al. (2009) (34)	2006-2007, Mymensingh^#^	315 251	All	N/A	111 (35.28) 33 (14.28)	PAGE-SS RT-PCR	RVA	N/A
Ahmed et al. (2010) (35)	2005-2006, Dhaka	656	<5 years	1.3:1	259 (39.5)	ELISA	RVA	N/A
Rahman et al. (2011) (36)	2006-2009, Dhaka	7058	All	N/A	1607 (23)	Dakopatts kit ELISA	RVA	N/A
Afrad et al. (2013) (37)	2006-2012, Matlab	9678	All	N/A	1936 (20.3)	ELISA	RVA	N/A
Sarker et al. (2014) (38)	Dhaka, 1993-1997	9879	<5 years	1.6:1	2493 (25)	ELISA	RVA	N/A
	Dhaka 2008-2012	6575			2864 (42)			
	Total	16454			5347 (32.5)			
Tanmoy et al. (2016) (39)	2012, Dhaka	297	<5 years	1.6:1	159 (54)	RT-PCR ELISA	RVA	N/A
Satter et al. (2017) (40)	2012-2015, Dhaka, Rajshahi, Sylhet, Chittagong, Rangpur, Khulna and Barisal	3783	<5 years	1.6:1	2432 (64)	ELISA RT-PCR	RVA	N/A
Haque et al. (2018) (41)	2012-16, Dhaka	1110	All	N/A	351 (32)	ELISA RT-PCR	RVA	N/A
Satter et al. (2018) (42)	2012-2017, Dhaka, Rajshahi, Sylhet, Chittagong, Rangpur, Khulna and Barisal	7562	<5 years	N/A	4,832 (64)	ELISA RT-PCR	RVA	N/A
Dey et al. (2020) (43)	2014-2019, Chattogram, Savar and Sirajganj	572	<15 years	1.6:1	211 (36.8)	RT-PCR	RVA	Norovirus, adenovirus, human bocavirus
Sharif et al. (2020) (44)	2014-2019, Chattogram, Savar and Sirajganj	387	<15	1.1:1	102 (26.3) (29.8)	RT-PCR	RVA	*E. coli* *Vibrio cholerae* *Salmonella* *Shigella* Norovirus, adenovirus, human bocavirus
Sarkar et al. (2019) (45)	2014*	82	All	N/A	50	RT-PCR	RVA	N/A
Islam et al. (2020) (46)	2013, Multiple site*	454	N/A	N/A	45 (10)	RT-PCR	RVA	N/A
Islam et al. (2020) (47)	2011-2014, Dhaka, Faridpur, Manikganj and Tangail, Rajbari*	416	N/A	N/A	50 (12)	RT-PCR	RVA	N/A
Hossain et al. (2020) (48)	2009-2010, Netrokona Dinajpur, Chattogram*	241	N/A	N/A	15 (6.2)	RT-PCR	RVA	N/A
		279	N/A	N/A	46 (16.5)			

*non-human primates.

#Birds

### Temporal and spatial distribution of rotavirus infection

**Figure 2:**
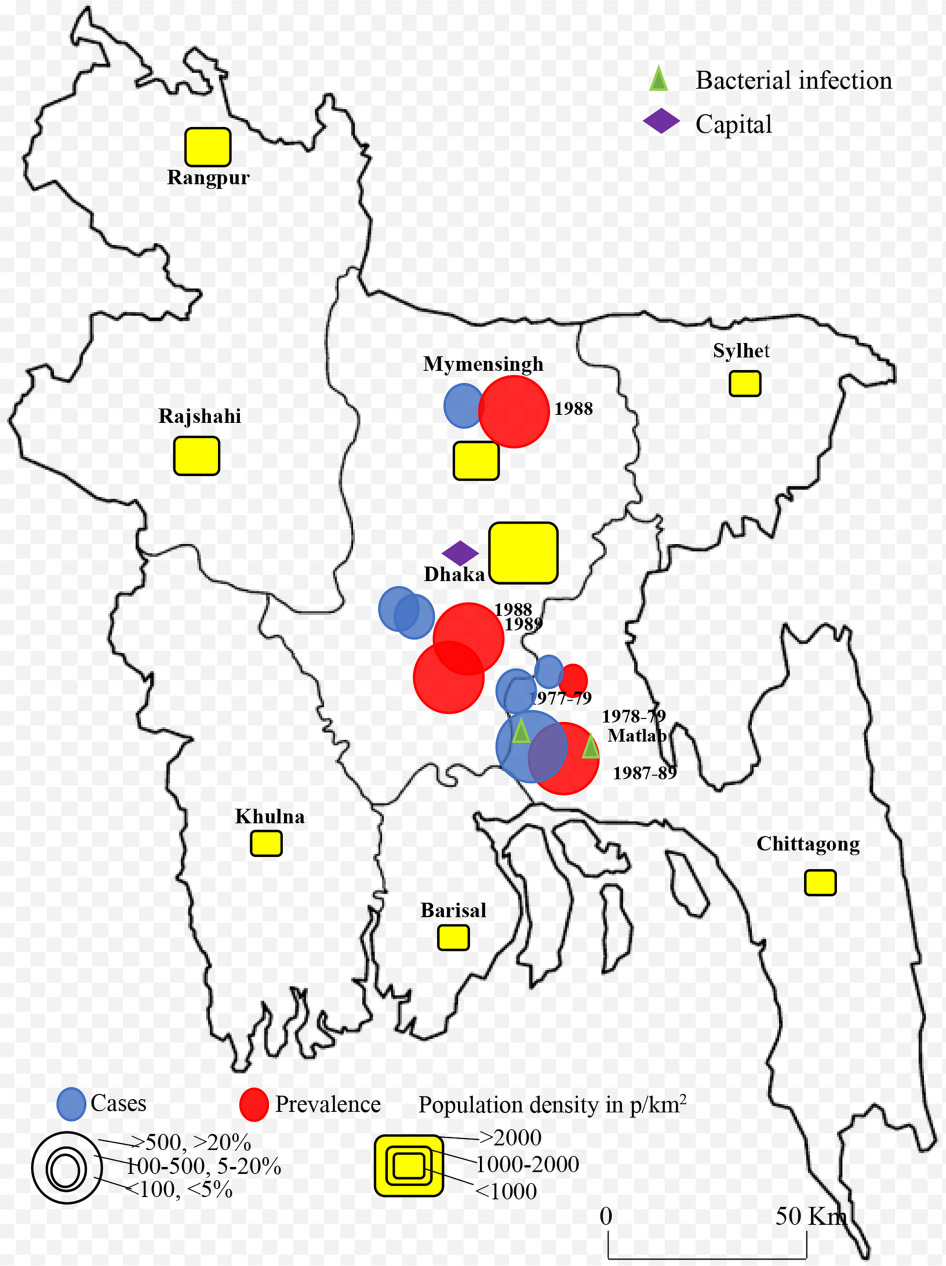
Spatial and temporal distribution of rotavirus cases and prevalence in Bangladesh during 1973-1999 and B. 2000-2023. Blue color circle indicated cases, red color circle indicated prevalence yellow color box indicated population density in p/km^2^.

**Figure 3:**
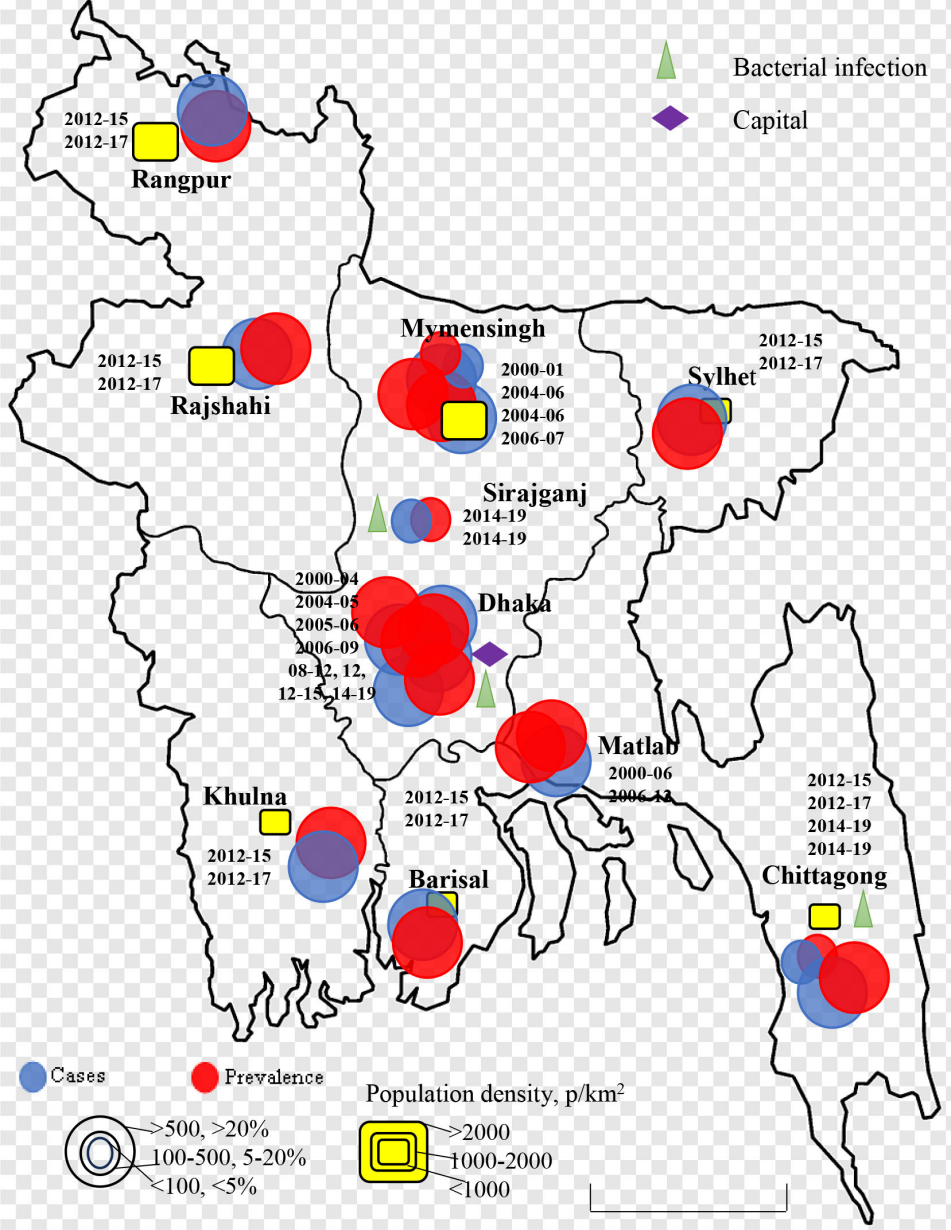
Spatial and temporal distribution of rotavirus cases and prevalence in Bangladesh during 2000-2023. Blue color circle indicated cases, red color circle indicated prevalence yellow color box indicated population density in p/km^2^.

Rotavirus infection was detected across the country. The first report was from Matlab, Chandpur during 1977-1979 with lower prevalence and bacterial co-infection (20). Only few studies were conducted in selected cities in Bangladesh during 1975-1999 (20–25). Larger prevalence (>20%) of rotavirus among children was detected in Matlab during 1978-1979 and 1987-1989 followed by Dhaka and Mymensingh during 1988 and 1989 (**Figure 2**). However, cases of rotavirus were not reported from any other part of the country during 1975-1999. The number of studies increased during 2000-2023 in Bangladesh. About 22 studies were conducted on the prevalence and epidemiology of rotavirus on human during 2000-2023 (**Figure 3**) (26–49). Majority of the studies were reported from the three cities including Dhaka, Mymensingh and Matlab until 2010 in Bangladesh. Two surveillance studies during 2012-2015 and 2012-2017 included rotavirus cases from 8 divisional cities in Bangladesh and reported higher prevalence of about 64% (**Figure 3**). Besides these divisional cities studies on rotavirus have been conducted only in limited areas including Sirajganj, Savar and Tangail. Presence of other enteric viruses and bacterial co-infection have been reported from Dhaka, Chittagong, Savar, Tangail, and Sirajganj during 2010-2019 (43, 44).

### Genetic diversity and distribution of rotavirus in Bangladesh

**Figure 4:**
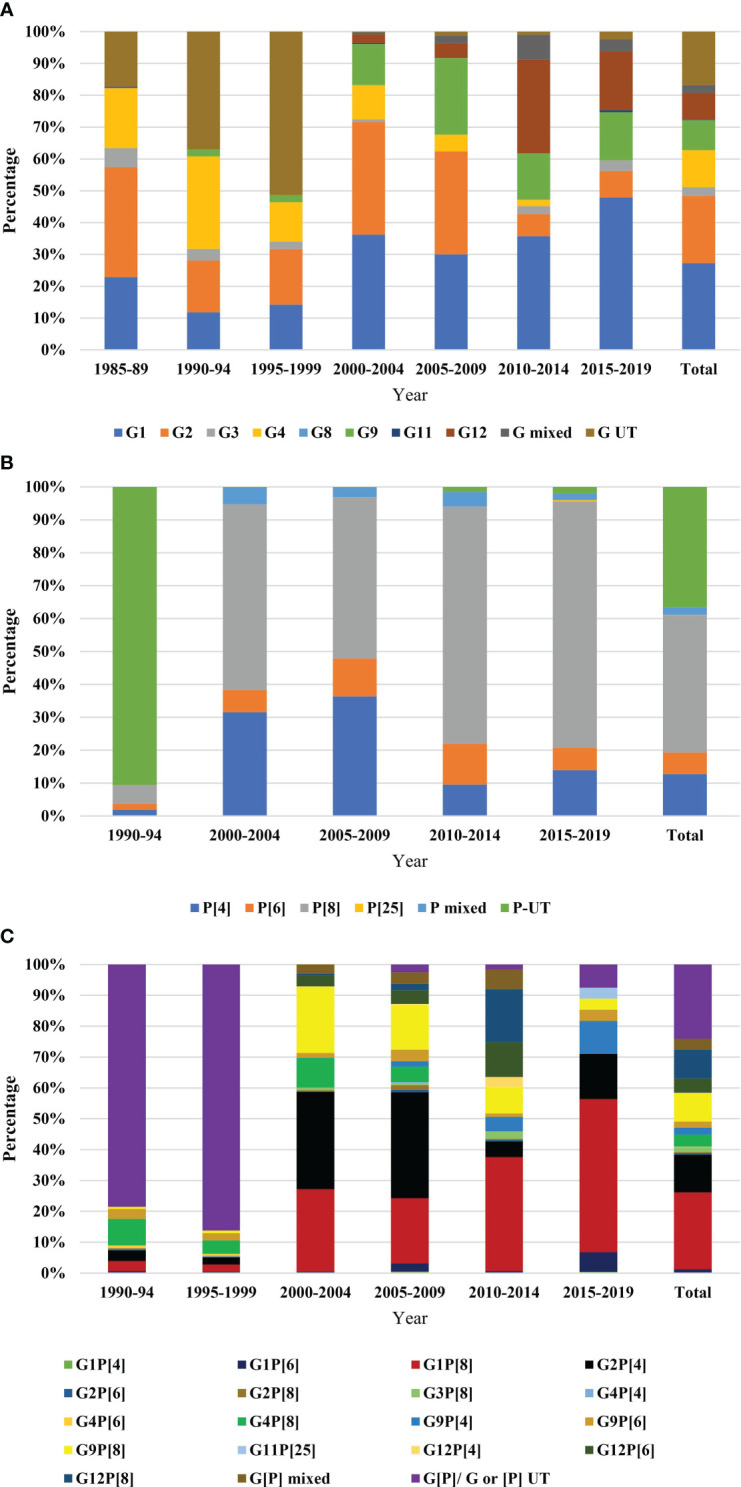
Year-wise genotypic distribution of rotavirus **(A)** G-type, data were represented from 1985 to 2019. Five years interval was used. The bar in the right side represented total diversity during 1985-2019, **(B)** P-type, data were represented from 1990 to 2019. Five years interval was used. The bar in the right side represented total diversity during 1990-2019, and **(C)** G[P]-types, data were represented from 1990 to 2019. Five years interval was used. The bar in the right side represented total diversity during 1990-2019.

Data on genetic diversity of rotavirus from all the reported works were included. Rotavirus G-typing was found in 19 articles, P-typing in 13 articles and G [P] typing in 13 articles. G-typing was conducted for 8219 samples. Rotavirus G1 (27.1%, 2228 of 8219) was the most prevalent followed by G2 (21.09%, 1733 of 8219), G4 (11.58%, 952 of 8219), G9 (9.37%, 770 of 8219), G12 (8.48%, 697 of 8219), and G3 (2.79%, 229 of 8219), respectively. About 2.5% (200 of 8219) of the isolates were mixed and 16.7% were non-typable G type (**Table 2A**). The number of studies on P typing were less than G typing. Among the reported P-type in Bangladesh, P[8] (40.6%, 2548 of 6274) was the most prevalent followed by P[4] (12.4%, 777 of 6274) and P[6] (6.4%, 400 of 6274), respectively. About 35.5% samples were non-typable and 2.3% were mixed P type in Bangladesh during 1973-2023 (**Table 2B**). Significantly diverse genotypes of rotavirus have been reported in Bangladesh. Among the regularly reported genotypes of rotavirus, G1P[8] (19.1%, 1199 of 6274) was the most frequent followed by G2P[4] (9.4%, 590 of 6274), G12P[8] (7.2%, 451 of 6274), G9P[8] (6.97%, 437 of 6274), G12P[6] (3.5%, 219 of 6274), G4P[8] (2.9%, 183 of 6274), G9P[4] (1.8%, 111 of 6274) and G9P[6] (1.5%, 94 of 6274), respectively (**Table 2C**). Other irregularly irregularly reported genotypes of rotavirus in Bangladesh included, G1P[4], G1P[6], G2P[6], G2P[8], G3P[3], G3P[15], G3P[8], G4P[4], G4P[6], G4P[49], G6P[11], G8P[1], G10P[1], G10P[11], G10P[15], G11P[25], and G12P[4] (**Table 2C**). Yearly distribution of genotypes of rotavirus showed significant changes during the study period. During 1985-1989, G2 (25%) was the most prevalent followed by G1 (22%) and G4 (19%) and during the next 10 years the prevalence of non-typable G type (45%) increased significantly. However, during 2000-04 and 2004-09, the prevalence of G1 (33%), G2 (32%) and G9 (16%) increased. In the next ten years (2010-2019), a sharp increase in the prevalence of G12 (18%) was observed and G1 (40%) remained the most prevalent genotype in Bangladesh. Prevalence of genotype G4 decreased from 20% to 2% from 1985 to 2019 (**Figure 4A**).

**Table 2A:** Spatial and temporal distribution of rotavirus G type in Bangladesh.

Study	Year/Place	Number of G typed	G1	G2	G3	G4	G8	G9	G11	G12	G mixed	G UT
Ahmed et al. (1989) (22)	1988, Dhaka, Mymensingh	51	15 (29.4)	20 (39.2)	1 (2)	9 (17.6)	N/A	N/A	N/A	N/A	6 (11.8)	N/A
Tabassum et al. (1989) (23)	1985-87, Dhaka	32	4 (12.5)	13 (40.6)	4 (12.5)	8 (25)	N/A	N/A	N/A	N/A	3 (9.4)	N/A
Ahmed et al. (1991) (51)	1988, Dhaka, Mymensingh	110	15 (13.6)	11 (10)	1 (0.9)	9 (8.2)	N/A	N/A	N/A	N/A	N/A	N/A
Fun et al. (1991) (24)	1987-89, Matlab	558	166 (29.7)	228 (40.9)	39 (7)	125 (22.4)	N/A	N/A	N/A	N/A	N/A	N/A
Berne et al. (1992) (25)	1987-89 Matlab	485	146 (30.1)	198 (40.8)	36 (7.4)	105 (21.6)	N/A	N/A	N/A	N/A	N/A	233
Unicomb et al. (1999) (49)	1992-97, Dhaka	2515	324 (12.9)	423 (16.8)	77 (3.1)	540 (21.5)	N/A	56 (2.2)	N/A	N/A	N/A	1095 (43.5)
Rahman et al. (2007) (27)	2001-2006, Dhaka Matlab	471	162 (34.4)	98 (20.8)	N/A	39 (8.3)	N/A	128 (27.2)	3 (0.6)	26 (5.5)	N/A	N/A
Paul et al. (2008) (30)	2004-2006, Mymensingh	155	35 (22.6)	81 (52.3)	N/A	3 (1.9)	N/A	22 (14.2)	N/A	5 (3.2)	7 (4.5)	2 (1.3)
Zaman et al. (2009) (32)	2000-2006, Matlab	406	207 (51)	125 (30.8)	N/A	50 (12.3)	N/A	–	N/A	20 (4.9)	N/A	N/A
Dey et al. (2009) (33)	2004-2005, Dhaka	307	39 (12.7)	132 (43)	8 (2.6)	60 (19.5)	N/A	42 (13.7)	N/A	–	N/A	N/A
Ahmed et al. (2009) (35)	2005-2006, Dhaka	145	36 (24.8)	66 (45.5)	N/A	3 (2.1)	N/A	12 (8.3)	N/A	14 (9.7)	2 (1.4)	12 (8.3)
Rahman et al. (2011) (36)	2006-2009, Dhaka	166	51 (30.7)	53 (31.9)	N/A	N/A	N/A	54 (32.5)	N/A	7 (4.2)	1 (0.6)	N/A
Afrad et al. (2013) (37)	2006-2012, Matlab	244	85 (34.8)	77 (31.6)	N/A	N/A	N/A	82 (33.6)	N/A	N/A	N/A	N/A
Afrad et al. (2013) (37)	2006-2012, Matlab	183	44 (24)	34 (18.6)	N/A	1 (0.5)	N/A	49 (26.8)	N/A	28 (15.3)	25 (13.7)	1 (0.5)
Tanmoy et al. (2016) (39)	2012, Dhaka	159	50 (31.4)	3 (1.9)	N/A	N/A	N/A	31 (19.5)	N/A	64 (40.3)	11 (6.9)	N/A
Satter et al. (2017) (40)	2012-2015, Dhaka, Rajshahi, and Sylhet, Chittagong and Rangpur, Khulna and Barisal	543	174 (32)	43 (8)	N/A	N/A	N/A	58 (11)	N/A	217 (40)	51 (9)	N/A
Haque et al. (2018) (41)	2012-16, Dhaka, hospitalized	351	104 (29.6)	24 (6.8)	14 (4)	N/A	N/A	57 (16.2)	N/A	73 (20.8)	N/A	N/A
Satter et al. (2018) (42)	2012-2017, Dhaka, Rajshahi, and Sylhet, Chittagong and Rangpur, Khulna and Barisal	1079	474 (43.9)	49 (4.5)	49 (4.5)	N/A	N/A	147 (13.6)	N/A	247 (22.9)	94 (8.7)	20 (1.9)
Dey et al. (2020) (43)	2014-19, Chattogram, Savar and	140	80 (57)	28 (20)	N/A	N/A	N/A	28 (20)	4 (3)	N/A	N/A	10 (7)
Paul et al. (2010) (31)	2004-2006, Mymensingh	60	16 (27)	27 (45)	N/A	N/A	N/A	4 (7)	N/A	6 (10)	N/A	N/A
Islam et al. (2020) (46)	2011-2014 Dhaka, Faridpur, Manikganj and Tangail, Rajbari	16	1 (6.3)	N/A	N/A	N/A	1 (6.3)	N/A	N/A	N/A	N/A	N/A
Sarkar et al. (2019) (45)	2014	36	N/A	N/A	N/A	36 (100)	N/A	N/A	N/A	N/A	N/A	N/A
Islam et al. (2020) (47)	2013	7	N/A	N/A	N/A	N/A	N/A	N/A	N/A	N/A	N/A	N/A
Total	–	8219	2228 (27.11)	1733 (21.09)	229 (2.79)	952 (11.58)	1 (0.01)	770 (9.37)	7 (0.09)	697 (8.48)	200 (2.43)	1373 (16.71)

**Table 2B:** Spatial and temporal distribution of rotavirus P type in Bangladesh.

Study	Year/Place	Number of P typed	P[4]	P[6]	P[8]	P[25]	P-mixed	P-UT
Unicomb et al. (1999) (49)	1992-97, Dhaka	2515	45 (1.8)	45 (1.8)	136 (5.4)	N/A	N/A	2180 (86.7)
Rahman et al. (2007) (28)	2001-2006, Dhaka, Matlab	471	95 (20.2)	35 (7.4)	326 (69.2)	N/A	N/A	N/A
Paul et al. (2008) (30)	2004-2006, Mymensingh	155	74 (47.7)	25 (16.1)	28 (18.1)	N/A	26 (16.8)	2 (1.3)
Dey et al. (2009) (33)	Dhaka, (2004-2005)	307	132 (42.9)	N/A	163 (53.2)	N/A	N/A	N/A
Ahmed et al. (2010) (35)	Dhaka, 2005-2006	145	67 (145)	19 (13.1)	42 (29)	N/A	12 (8.3)	4 (2.8)
Paul et al. (2010) (31)	Mymensingh, 2004-2006	60	43 (71.7)	12 (20)	20 (33.3)	N/A	23 (38.3)	N/A
Rahman et al. (2011) (36)	Dhaka, 2006-2009	166	49 (29.5)	20 (12)	93 (56)	N/A	4 (2.4)	N/A
Afrad et al. (2013) (37)	Matlab, 2006-2012	183	42 (23)	18 (9.8)	101 (55.2)	N/A	4 (2.2)	1 (0.5)
Tanmoy et al. (2016) (39)	2012, Dhaka	159	14 (8.8)	20 (12.6)	123 (77.4)	N/A	11 (6.9)	2 (1.3)
Satter et al. (2017) (40)	2012-2015, Dhaka, Rajshahi, Sylhet, Chittagong, Rangpur, Khulna and Barisal	543	41 (7)	63 (12)	410 (76)	N/A	24 (4)	5 (1)
Haque et al. (2018) (41)	2012-16, Dhaka	351	53 (15.1)	26 (7.4)	192 (54.7)	N/A	N/A	11 (3.1)
Satter et al. (2018) (42)	2012-2017, Dhaka, Rajshahi, Sylhet, Chittagong, Rangpur, Khulna and Barisal	1079	80 (7.4)	97 (9)	850 (78.8)	N/A	38 (3.5)	14 (1.3)
Dey et al. (2020) (43)	2014-19, Chattogram, Savar	140	42 (30)	20 (14)	64 (46)	4 (3)	N/A	10
Total	–	6274	777 (12.4)	400 (6.4)	2548 (40.6)	4 (0.1)	142 (2.3)	2229 (35.5)

**Table 2C:** Spatial and temporal distribution of rotavirus genotypes in Bangladesh during 1973-2023.

Study	Year/Place	Number of G[P]typed	G1P[4]	G1P [6]	G1P [8]	G2P [4]	G2P [6]	G2P [8]	G3P [8]	G4P [4]	G4P [6]	G4P [8]	G9P [4]	G9P [6]	G9P [8]	G11P [25]	G12P [4]	G12P[6]	G12P[8]	GP mixed	GP/G or P UT
Unicomb et al. (1999) (49)	1992-97, Dhaka	2515	2 (.1)	5 (.2)	37 (1.5)	38 (1.5)	3 (0.1)	3 (0.1)	N/A	3 (0.1)	9 (0.4)	85 (3.4)	N/A	37 (1.5)	10 (0.4)	N/A	N/A	N/A	N/A	N/A	1095 (43.5)
Rahman et al. (2007) (28)	2001-2006, Dhaka Matlab	471	N/A	3 (0.6)	159 (33.8)	95 (20.2)	1 (0.2)	2 (0.4)	N/A	N/A	N/A	39 (8.3)	N/A	9 (1.9)	119 (25.3)	N/A	N/A	21 (4.5)	5 (1.1)	15 (3.2)	N/A
Paul et al. (2008) (30)	2004-2006, Mymensingh	155	N/A	9 (5.8)	16 (10.3)	70 (45.2)	3 (1.9)	1 (0.6)	N/A	1 (0.6)	N/A	1 (0.6)	1 (0.6)	5 (3.2)	9 (5.8)	N/A	1 (0.6)	4 (2.6)	N/A	4 (2.6)	N/A
Dey et al. (2009) (33)	2004-2005, Dhaka	307	N/A	N/A	36 (11.8)	120 (39)	N/A	N/A	9 (2.9)	N/A	N/A	56 (18.2)	N/A	N/A	40 (13)	N/A	N/A	N/A	N/A	N/A	N/A
Ahmed et al. (2010) (35)	2005-2006, Dhaka	145	3 (2.1)	2 (1.4)	24 (16.6)	57 (39.3)	1 (0.7)	3 (2.1)	N/A	2 (5.5)	N/A	1 (8.3)	3 (1.4)	8 (5.5)	N/A	N/A	N/A	12 (8.3)	2 (1.4)	N/A	15 (10.3)
Paul et al. (2010) (31)	2004-2006, Mymensingh	60	N/A	N/A	6 (10)	22 (36.7)	N/A	N/A	N/A	N/A	N/A	N/A	N/A	N/A	4 (6.7)	N/A	1 (1.7)	2 (3.4)	N/A	7 (11)	N/A
Rahman et al. (2011) (36)	Dhaka 2006-2009	166	N/A	3 (1.8)	46 (27.7)	47 (28.3)	1 (0.6)	3 (1.8)	N/A	N/A	N/A	N/A	2 (1.2)	9 (5.4)	43 (25.9)	N/A	N/A	7 (4.2)	N/A	N/A	N/A
Afrad et al. (2013) (37)	2006-2012, Matlab	183	N/A	3 (1.6)	41 (22.4)	31 (16.9)	1 (0.5)	2 (1.1)	N/A	N/A	N/A	1 (0.5)	7 (3.8)	4 (2.2)	38 (20.8)	N/A	1 (0.5)	8 (4.4)	19 (10.4)	26 (14.2)	1 (0.5)
Tanmoy et al. (2016) (39)	2012, Dhaka	159	N/A	4 (2.5)	46 (28.9)	3 (1.9)	N/A	N/A	N/A	N/A	N/A	N/A	10 (6.3)	8 (5)	13 (8.2)	N/A	N/A	7 (4.4)	55 (34.6)	11 (6.9)	2 (1.3)
Satter et al. (2017) (40)	2012-2015, Dhaka, Rajshahi, Sylhet, Chittagong, Rangpur, Khulna and Barisal	543	N/A	N/A	169 (31)	34 (6.3)	3 (0.6)	1 (0.2)	N/A	N/A	N/A	N/A	4 (0.7)	2 (0.4)	44 (8.1)	N/A	1 (0.2)	57 (10.5)	158 (29.1)	9 (1.7)	5 (0.9)
Haque et al. (2018) (41)	2012-16 Dhaka	351	2 (0.6)	2 (0.6)	95 (27.1)	11 (3.1)	3 (0.9)	3 (0.9)	14 (4)	N/A	N/A	N/A	31 (8.8)	4 (1.1)	17 (4.8)	N/A	2 (0.6)	17 (4.8)	53 (15.1)	79 (22.5)	11 (3.1)
Satter et al. (2018) (42)	2012-2017 Dhaka, Rajshahi, Sylhet, Chittagong, Rangpur, Khulna and Barisal	1079	1 (0.1)	1 (0.1)	464 (43)	37 (3.4)	6 (0.6)	1 (0.1)	48 (4.4)	N/A	N/A	N/A	36 (3.3)	4 (0.4)	96 (8.9)	N/A	2 (0.2)	84 (7.8)	159 (14.7)	18 (1.7)	20 (1.9)
Dey et al. (2020) (43)	2014-19, Chattogram, Savar	140	N/A	16 (11)	60 (43)	25 (18)	N/A	N/A	N/A	N/A	N/A	N/A	17 (12)	4 (3)	4 (3)	4 (3)	N/A	N/A	N/A	N/A	10 (7.1)
Total	–	6274	8 (0.13)	48 (0.77)	1199 (19.11)	590 (9.4)	22 (0.35)	19 (0.30)	71 (1.1)	6 (0.1)	9 (0.14)	183 (2.9)	111 (1.8)	94 (1.5)	437 (6.97)	4 (0.06)	8 (0.13)	219 (3.5)	451 (7.2)	169 (2.7)	1159 (18.47)

Majority of the isolates were un-typable (90%) for P-type during 1990-94. The prevalence of P[8] rotavirus increased from 7% to 72% during 1990 to 2019 and remained the most reported P type in Bangladesh. A sharp decrease in the prevalence of P[4] from 31% to 14% was reported from 2000 to 2019 (**Figure 4B**).

Among the circulating strains, 80-90% were un-typable during 1990-1999. Genotype G4P[8] was prevalent (7%) during 1990-99, the prevalence increased in 2000-05 (10%) and decreased to 1% during 2005-2019. During 2000-04, genotype G2P [4] (32%) was the most prevalent followed by G1P[8] (28%), G9P[8] (23%) and G4P[8] (10%), respectively. During the next five years (2005-09), the prevalence of G2P[4] increased to 38%. After 2009, genotype G1P[8] (50%) became the most predominant and the diversity of genotypes increased by 80% during 2010-2019 (**Figure 4C**).

Regional distribution of rotavirus revealed that G1 was most prevalent in Dhaka (42.3%, 942 of 2228) followed by Matlab (38%, 848 of 2228) and Mymensingh (5.9%, 132 of 2228), respectively. Genotype G2 and G4 were also most prevalent in Dhaka (48.7% and 69%, respectively) and Matlab (39% and 28%, respectively). Diversity of G genotype was significantly higher in Dhaka than any other cities in Bangladesh. Among the sequenced P types, P[4], P[6] and P[8] were also prevalent in Dhaka (about 50%) followed by Matlab (about 30%) and Mymensingh (about 10%). About 45% (95% CI: 32-63%) of reported G1P[8], G2P[4], G12P[8], and G9P[8] were documented in Dhaka, followed by 31% (95% CI: 22-43%) in Matlab. Genotypes G1P[8], G2P[4], G2P[6], G9P[4] G12P[8], and G9P[8] were also reported in minor frequency from Mymensingh, Rajshahi, Sylhet, Chittagong, Rangpur, Khulna and Barisal (**Table 2C**).

### Clinical characteristics of patients with rotavirus

**Figure 5:**
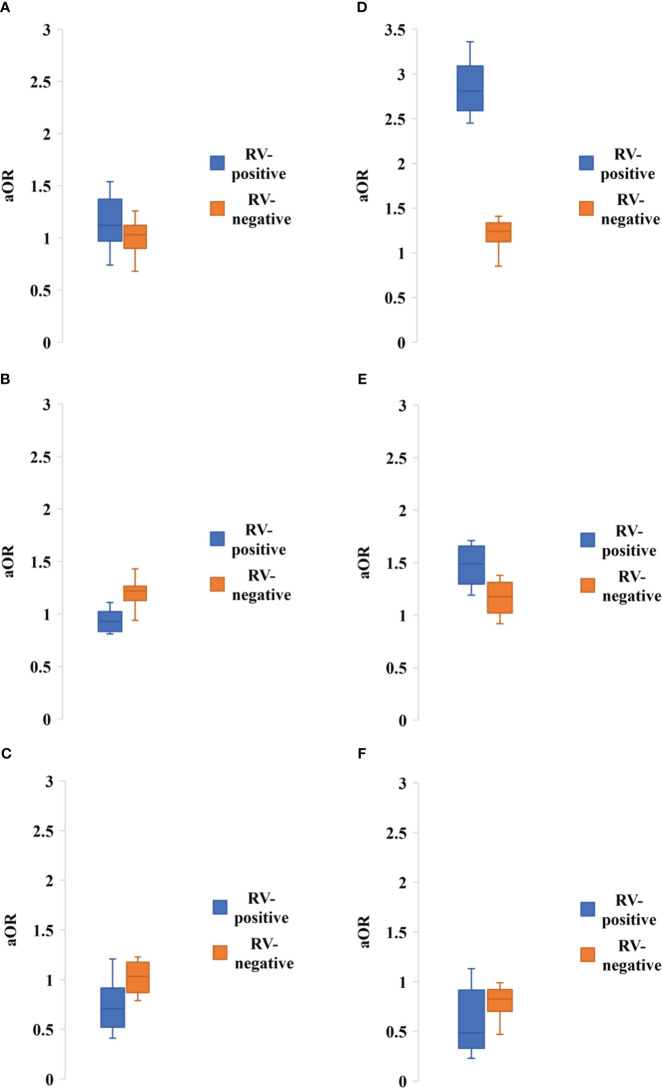
Adjusted odds ratio of having symptoms of **(A)** Diarrhea, **(B)** Severe dehydration, **(C)** Fever, **(D)** Watery stool, **(E)** Abdominal pain, **(F)** Hospitalization among people with rotavirus positive vs negative infection. Blue bar indicated rotavirus positive and brown bar indicated rotavirus negative cases.

About 19 of the studies included clinical symptoms of rotavirus infected people. Diarrhea was the most common symptoms followed by dehydration, vomiting, fever and abdominal pain, respectively. About 89.5% (17 of 19) of the studies reported diarrhea followed by dehydration (63.1, 12 of 19), vomiting (57.9%, 11 of 19), fever (57.9%, 11 of 19) and abdominal pain (36.8, 7 of 19), respectively (**Table 3**). Hospitalization was required for 83% (95% CI: 62-98%, *p* = 0.001) of the reported cases of rotavirus in Bangladesh. Diarrhea (97.3%, 95% CI: 87-99%, *p* = 0.04) with characteristics watery stools of rotavirus infection (70.5%, 95% CI: 63-89%, *p*=0.005) was the most prevalent symptoms followed by vomiting (56.5%, 95% CI: 45-89%, *p* = 0.006), dehydration (28.8%, 95% CI: 24-75%, *p* = 0.002), and fever (16.5%, 95% CI: 8-54%, *p* = 0.003), respectively (**Table 3**). Only three of the studies reported death of children infected with rotavirus with mortality rate varying from 0.1-1.6%. Rotavirus positive children had higher risk of developing severe dehydration (aOR: 2.81, 95% CI: 2.45-3.36, *p* = 0.005), diarrhea (aOR: 1.12, 95% CI: 0.74-1.54, *p* = 0.01), and watery stool (aOR: 1.49, 95% CI: 1.19-1.71, *p* = 0.003) (**Figure 5**). However, rotavirus negative children with diarrhea had higher risk of developing fever (aOR: 1.22, 95% CI: 0.94-1.43, *p* = 0.005) and abdominal pain (aOR: 1.03, 95% CI: 0.79-1.23, *p* = 0.01) (**Figure 5**).

**Table 3:** Clinical symptoms of rotavirus infection among children in Bangladesh during 1973-2023.

Study	RV-positives	Symptoms						Hospitalization	Death
		Dehydration	Vomiting	Fever	AP	WS	Diarrhea		
Black et al. (1981) (21)	43	43 (100)	N/A	N/A	N/A	43 (100)	43 (100)	30 (69.8)	N/A
Tabassum et al. (1989) (23)	111	N/A	N/A	N/A	N/A	111 (100)	111 (100)	111 (100)	N/A
Fun et al. (1991) (24)	898	N/A	N/A	N/A	N/A	845 (94)	898 (100)	N/A	N/A
Berne et al. (1992) (25)	764	756 (99)	710 (93)	91 (12)	N/A	749 (98)	764 (100)	N/A	12 (1.6)
Sanekata et al. (2003) (26)	14^* b^	14 (100)	14 (100)	7 (50)	0 (0)	11 (78.6)	N/A	14 (100)	N/A
Rahman et al. (2005) (27)	14^# c^	11 (78.6)	13 (93)	1 (7.1)	7 (50)	14 (100)	N/A	14 (100)	N/A
Paul et al. (2008) (30)	522	N/A	N/A	N/A	N/A	N/A	100	100	N/A
Zaman et al. (2009) (32)	1304	261 (20.3)	1108 (85)	1030 (79)	N/A	1173 (90)	1304 (100)	847 (65)	N/A
Dey et al. (2009) (33)	9	9 (100)	5 (55)	3 (33.3)	7 (77.8)	9 (100)	9 (100)	9 (100)	N/A
Islam et al. (2009) (34)	111	N/A	N/A	N/A	N/A	N/A	111 (100)	111 (100)	N/A
Selim et al. (2009) (50)	252	106 (43)	164 (65)	147 (58.4)	N/A	252 (100)	252 (100)	162 (64)	2 (0.8)
Ahmed et al. (2010) (35)	259	N/A	N/A	N/A	N/A	N/A	259 (100)	259 (100)	N/A
Rahman et al. (2011) (36)	1607	N/A	N/A	N/A	N/A	N/A	1607 (100)	1607 (100)	N/A
Afrad et al. (2013) (37)	1963	N/A	N/A	N/A	N/A	N/A	1963 (100)	1963 (100)	N/A
Sarker et al. (2014) (38)	2493	457 (18)	2164 (87)	262 (10)	491 (20)	2408 (97)	2493 (100)	1048 (43)	N/A
	2864	985 (34)	2125 (74)	233 (8)	463 (16)	2796 (98)	2864 (100)	1196 (42)	N/A
Tanmoy et al. (2016) (39)	159	92 (58)	65 (41)	25 (16)	86 (54)	159 (100)	159 (100)	159 (100)	N/A
Satter et al. (2017) (40)	2432	1614 (67)	2432 (100)	599 (51)	0	2432 (100)	2432 (100)	2432 (100)	N/A
Haque et al. (2018) (41)	351	169 (48.1)	257 (73.2)	144 (41)	146 (41.6)	322 (91.7)	351 (100)	N/A	N/A
Dey et al. (2020) (43)	211	186 (88.7)	178 (84.3)	164 (77.8)	141 (67.1)	211 (100)	192 (91)	211 (100)	N/A
Total	16353	4703 (28.8)	9235 (56.5)	2706 (16.5)	1341 (8.2)	11535 (70.5)	15912 (97.3)	10273 (62.8)	14 (0.1)

*non-human primates; #Birds; ^b^rotavirus B; ^c^rotavirus C.

### Seasonality of rotavirus infection

**Figure 6:**
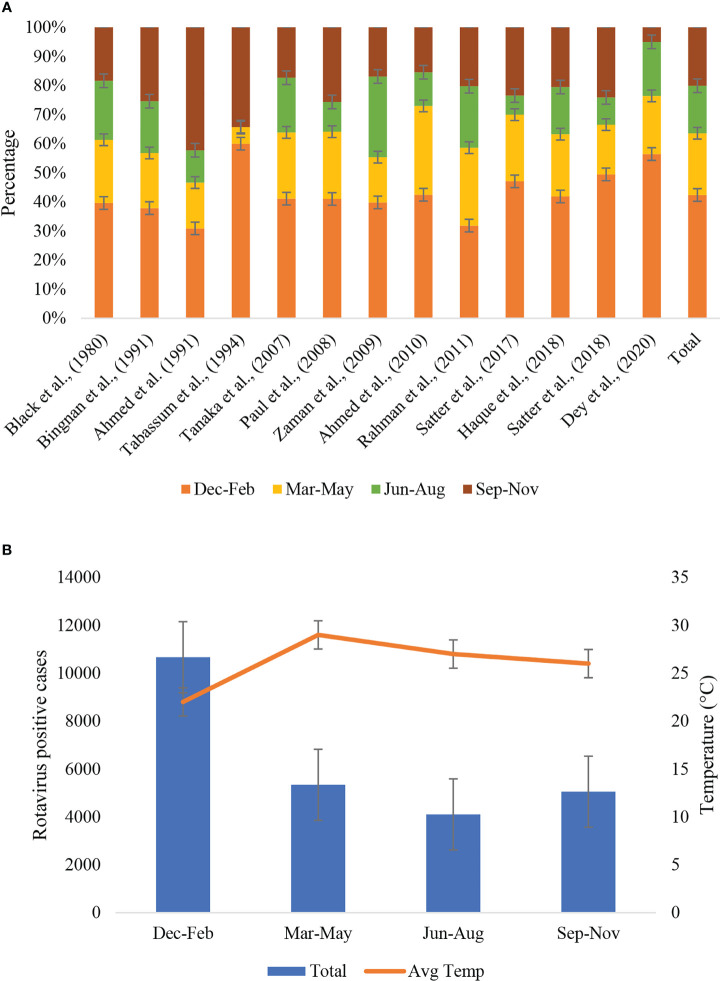
**(A)** Seasonal distribution of rotavirus infection described in different studies during 1973-2023 in Bangladesh. According to the variation of average temperature, four seasonal groups were included, December to February, March to May, June to August and September to November. The last bar in the right side represented cumulative seasonality of rotavirus infection. **(B)** Seasonal distribution of rotavirus positive cases and trends of average temperature in Bangladesh during 1973-2023.

**Figure 7:**
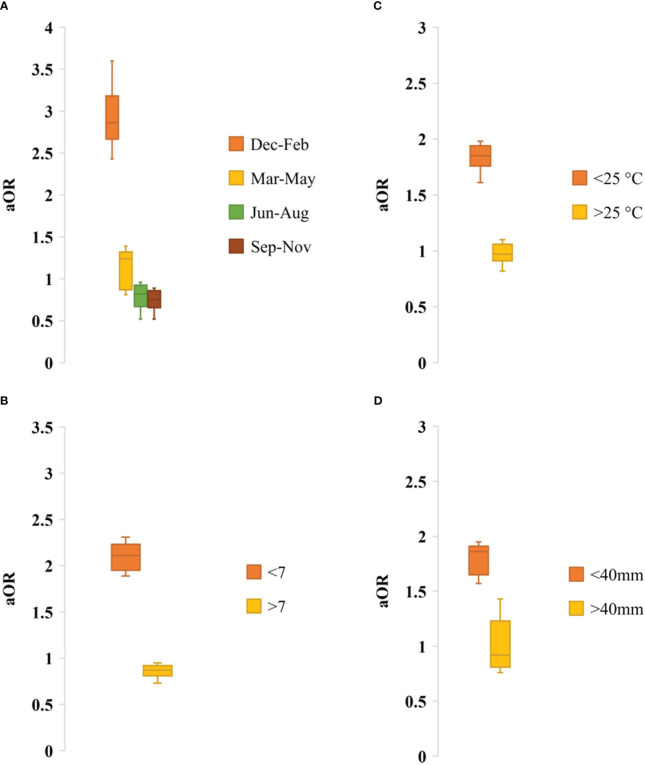
Adjusted odds ratio of rotavirus infection by **(A)** Seasonality, **(B)** UV index, **(C)** Average temperature and **(D)** Average rainfall.

Seasonality data of rotavirus infection was extracted from 13 studies during 1978-2023 in Bangladesh (**Figure 6**). Rotavirus infection in the study regions was detected all around the year. The highest peak of infection (43%, 95% CI: 39-58%, *p* = 0.001) was reported during December to February (winter season with average temperature 19-21°C) followed by March to May (21%, 95% CI: 17-31%, *p* = 0.002) (**Figure 6**). The pattern of the seasonal peak was consistent in all the included studies (**Table 4**). Impact of different environmental factors on the rotavirus incidence was determined. Incidence of rotavirus had higher odds of occurrence during December and February (aOR: 2.86, 95% CI: 2.43-3.6, *p* = 0.001) (**Figure 7**). We also found that higher odds of rotavirus infection at average temperature <25°C (aOR: 1.85, 95% CI: 1.61-1.94, *p* = 0.002), average UV index <7 (aOR: 2.11, 95% CI: 1.89-2.31, *p* = 0.004) and rainfall <40 mm (aOR: 1.86, 95% CI: 1.57-1.91, *p* = 0.005) (**Figure 7**).

**Table 4:** Month-wise proportionate distribution of rotavirus cases during 1973-2023 in Bangladesh.

Study	RV-positive	Dec-Feb	Mar-May	Jun-Aug	Sep-Nov
Black et al. (1980) (21)	2070	820 (39.6)	450 (21.7)	420 (20.3)	380 (18.4)
Fun et al. (1991) (24)	898	340 (37.9)	170 (18.9)	160 (17.8)	228 (25.4)
Ahmed et al. (1991) (51)	343	106 (30.9)	54 (15.7)	38 (11.1)	145 (42.3)
Tabassum et al. (1994) (23)	35	21 (60)	2 (5.7)	0 (0)	12 (34.3)
Tanaka et al. (2007) (29)	10683	4393 (41.1)	2430 (22.7)	2010 (18.8)	1850 (17.3)
Paul et al. (2008) (30)	39	16 (41)	9 (23.1)	4 (10.3)	10 (25.6)
Zaman et al. (2009) (32)	1479	589 (39.8)	230 (15.6)	410 (27.7)	250 (16.9)
Ahmed et al. (2010) (35)	259	110 (42.5)	79 (30.5)	30 (11.6)	40 (15.4)
Rahman et al. (2011) (36)	1607	512 (31.9)	430 (26.8)	340 (21.2)	325 (20.2)
Satter et al. (2017) (40)	2432	1145 (47.1)	557 (22.9)	160 (6.6)	570 (23.4)
Haque et al. (2018) (41)	351	147 (41.9)	75 (21.4)	57 (16.2)	72 (20.5)
Satter et al. (2018) (42)	4832	2390 (49.5)	827 (17.1)	450 (9.3)	1165 (24.1)
Dey et al. (2020) (43)	140	79 (56.4)	28 (20)	26 (18.6)	7 (5)
Total	25168	10668 (42.4)	5341 (21.2)	4105 (16.3)	5054 (20.1)

### Risk factors of rotavirus infection in Bangladesh

**Figure 8:**
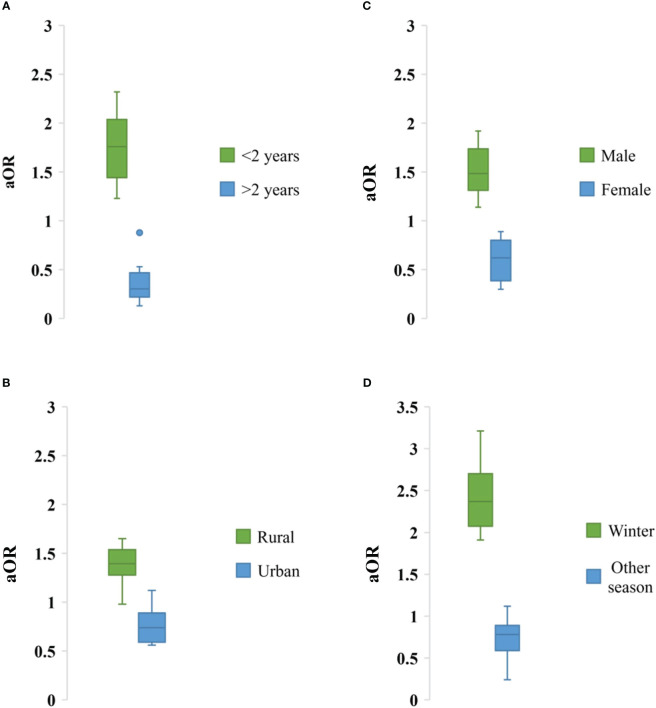
Association of different sociodemographic factors **(A)** Age, **(B)** Residence, **(C)** Sex and **(D)** Weather with odds of rotavirus infection in Bangladesh during 1973-2023.

Impact of socio-demographic factors associated with rotavirus infection was evaluated. Children aged <2 years had higher risk of rotavirus infection (aOR: 1.76, 95% CI: 1.23-2.32, *p* = 0.001), male children had higher risk of rotavirus infection (aOR: 1.48, 95% CI: 1.14-1.92, *p* = 0.003), children in rural areas had higher risk of infection (aOR: 1.4, 95% CI: 0.98-1.65, *p* = 0.01) than in urban areas in Bangladesh. In the seasonal analysis, we also found that rotavirus infection was prevalent during winter (aOR: 2.37, 95% CI: 1.91-3.21, *p* = 0.02) than other seasons (**Figure 8**).

## Discussion

Rotavirus has remained one of the major causes of children mortality and hospitalization in Bangladesh (43–45). However, rotavirus vaccination has not been included in the national immunization program in Bangladesh (9). As a result, the prevalence of rotavirus among children in Bangladesh is still high (40, 41, 43, 44). Before inclusion of rotavirus vaccine on national immunization program, integrated knowledge on prevalence, genotypic diversity, symptoms and seasonality of rotavirus can contribute significantly in the public health policy making. However, there is a lack of integrated study on rotavirus in Bangladesh. We conducted this study to provide an integrated insight of rotavirus infection in Bangladesh during pre-vaccination period. This is the first report of meta-analysis of rotavirus in Bangladesh including all data from 1973 to 2023. We found high prevalence of rotavirus (30.1%, 95% CI: 22%-45%, *p* = 0.005) in Bangladesh, that is higher than previous meta-analysis and surveillance study conducted in Turkey, China, worldwide data (50–65). However, a meta-study found higher prevalence of rotavirus in Iran. Prevalence of rotavirus ranged from 2%-64% in the included studies in Bangladesh. Further, we found higher prevalence of rotavirus among children aged <5 years (38.2%, 95% CI: 24%-51%, *p* = 0.001) than any other meta studies conducted previously (55, 56, 58–69). These data suggest that prevalence of rotavirus is relatively higher than most of the countries and a consistent health problem in Bangladesh (40–45, 65–72). This study also suggested a higher prevalence of rotavirus among people of all age groups. Other epidemiological findings including male to female ratio of (male was mostly infected) rotavirus infection was in good agreement with previous studies in Bangladesh, China, Turkey, India and Pakistan (50–56, 62–69). Most of the studies reported circulation of rotavirus A (99.9% cases) among Bangladeshi population, which is in good agreement with previous studies (60–69).

Recent studies after 2005 found higher prevalence (ranging from 23% to 64%) of rotavirus among children under 5 years in Bangladesh than previous studies. Usage of reverse transcriptase polymerase chain reaction and molecular sequencing were applied more commonly after 2000 in Bangladesh for the detection and molecular characterization of rotavirus. Only six of the studies reported co-circulation of bacterial pathogens among diarrheal children with rotavirus in Bangladesh. Among the reported coinfecting bacterial pathogens, *V. cholerae*, *Shigella* spp, and *Salmonella* spp were most commonly found in different studies with prevalence ranging from 1% to 32%. These findings are highly similar with previous studies in Bangladesh (43, 44, 70–72). We found high genotypic diversity of rotavirus A in Bangladesh. About 15 G-types and 8 P-types of rotaviruses have been documented. Genotype G1, G2 and G4 (60% combinedly) were the most prevalent rotaviruses in Bangladesh. Among the P-genotypes P[8] was the most frequent (40%) followed by P[4] (12%). These findings are in good agreement with pre-vaccination data in developing and developed countries (51, 55, 58–69). As mass vaccination is yet to be introduced, the prevalence and genotypic diversity remained unchanged over the years and places in Bangladesh. Further, genotypic diversity and heterogeneity due to mutation and recombination contributing to numerous un-typable G-types and P-types, which can contribute in lowering the vaccine efficacy in Bangladesh. Among the typable isolates, 28 genotypic combinations were documented, which is higher than the previous reports from a country (58–69). Rotavirus G1P[8] (19%) was the most prevalent and G2P[4], G9P[8] and G12P[8] were regularly reported among children with diarrhea in Bangladesh since 1978. This genotypic distribution is in good agreement with reported genotypic diversity data in Ethiopia, India, Iran and South Korea (51, 53–68). However, we detected relatively lower prevalence of G3 (2.8%) and G4 (11.6%) in Bangladesh than in other countries (60–68). This pattern of genotypic distribution reflects lack of vaccination contributed to the continuous prevalence of G1, G2, G9 and G12 in Bangladesh. These findings are highly similar with previous studies (51, 53–68).

We also found that only limited studies were conducted in Dhaka, Mymensingh and Matlab before 2000 in Bangladesh. The number of studies and localities increased during 2000-2010. Majority of the studies reported rotavirus from Dhaka, Matlab, Mymensingh (91%) and extended to Sylhet, Chittagong, Rangpur, Khulna and Barisal during 2010-2023. This study reports that published documents represent mostly hospitalized patients in selected areas (20–50). In future, more studies including samples from both home staying and hospitalized children with diarrhea from all over the country should be conducted. Hospital oriented surveillance during 2012-2017 included divisional cities and large number of samples. Molecular surveillance is lacking significantly to represent actual diversity of rotavirus in Bangladesh.

Clinical symptoms of rotavirus positive people were similar with previous reports (40–44). Diarrhea was the most prevalent symptoms followed by dehydration and vomiting. We found fever and abdominal pain inconsistently reported from the infected children in Bangladesh. Studies reporting co-infection of other bacterial pathogens have found higher frequency of fever, abdominal pain and vomiting than only rotavirus infection. Hospitalization was required for majority of the rotavirus infected children (80-100%) in Bangladesh. These findings are in good agreement with previous studies in Bangladesh (40–44).

We found significant impact of environmental factors and seasonality on rotavirus infection in Bangladesh. Rotavirus infection was detected all around the year with a sharp increase of cases during the winter season (December to February) with average air temperature 19-21°C. Further, we found that risk of rotavirus infection was high at lower temperature, lower UV index and lower amount of rainfall. These findings reflect seasonality and association of increased rotavirus infection with environmental parameters in Bangladesh (21, 23, 24, 29, 30, 32, 35, 36, 40–44). In some of the previous studies two peaks of rotavirus, one in winter and another in summer have been reported (40–44). However, in this integrated study we found a significant frequency of cases (about 48%) were documented during winter. Our findings are completely supported by previous studies in the developing countries. Previous studies have reported abundance of rotavirus infection among male children aged <5 years. However, lack of statistical analysis to evaluate the relationship between socio-demographic factors and rotavirus infection were found. In this study, we found that male (aOR: 1.48, 95% CI: 1.14-1.92, *p* = 0.003) children aged <2 years (aOR: 1.76, 95% CI: 1.23-2.32, *p* = 0.001) living in rural areas had higher risk of rotavirus infection in Bangladesh. These findings will add knowledge on the impact of sociodemographic factors on rotavirus infection. Our findings are supported strongly by previous findings (40–44, 51, 53–68).

The main limitation of this study was not including any data on rotavirus vaccination due to lack of studies. Further, we could not include mutational data of Bangladeshi rotavirus strains due to lack of studies. This study could be improved if data from all the regions were available and included for longer periods.

## Conclusions

This is the first meta-analysis including all the studies on prevalence, molecular epidemiology, and genetic diversity of rotavirus from 1973 to 2023 in Bangladesh. This study created an integrated overview of rotavirus infection in Bangladesh during 1973 to 2023. Vaccine coverage of rotavirus is still low in Bangladesh as rotavirus vaccines are not included in the EPI program. As a result, high prevalence of rotavirus (30.1%, 95% CI: 22%-45%, *p* = 0.005) in Bangladesh contributing to majority of health problems and mortality among children aged <5 years till now. We also found great genetic diversity of rotaviruses in the study regions with high prevalence of G1P[8], G2P[4], G9P[8] and G12P[8]. Introduction of existing rotavirus vaccines and development of more effective vaccines to prevent the health burden associated with rotavirus infection are immediately needed. This study will provide overall scenario of rotavirus infection during pre-vaccination period and aid in policy making for rotavirus vaccination program in Bangladesh.

## Data availability statement

The original contributions presented in the study are included in the article/supplementary material. Further inquiries can be directed to the corresponding authors.

## Author contributions

NadS: Conceptualization, Formal Analysis, Funding acquisition, Investigation, Methodology, Project administration, Resources, Software, Supervision, Validation, Visualization, Writing – original draft, Writing – review & editing. NazS: Formal Analysis, Investigation, Methodology, Software, Writing – review & editing. AK: Data curation, Formal Analysis, Investigation, Software, Writing – review & editing. IA: Data curation, Formal Analysis, Investigation, Software, Writing – review & editing. RM: Data curation, Formal Analysis, Investigation, Software, Writing – review & editing. ID: Data curation, Funding acquisition, Investigation, Resources, Software, Validation, Visualization, Writing – review & editing. AP: Data curation, Investigation, Methodology, Software, Writing – review & editing. SD: Conceptualization, Data curation, Investigation, Methodology, Project administration, Software, Supervision, Writing – review & editing.
